# Effects of attentional focus strategies in drop landing biomechanics of individuals with unilateral functional ankle instability

**DOI:** 10.3389/fphys.2024.1444782

**Published:** 2024-08-20

**Authors:** Zilong Wang, Lingyue Meng, Mengya Lu, Lingyu Kong, Jingxian Xue, Zhiqi Zhang, Xin Meng, Qiuxia Zhang

**Affiliations:** School of Physical Education, Soochow University, Suzhou, China

**Keywords:** functional ankle instability, attentional focus strategies, biomechanical characteristics, landing movements, motor control

## Abstract

**Background:**

Functional Ankle Instability (FAI) is a pervasive condition that can emerge following inadequate management of lateral ankle sprains. It is hallmarked by chronic joint instability and a subsequent deterioration in physical performance. The modulation of motor patterns through attentional focus is a well-established concept in the realm of motor learning and performance optimization. However, the precise manner in which attentional focus can rehabilitate or refine movement patterns in individuals with FAI remains to be fully elucidated.

**Objective:**

The primary aim of this study was to evaluate the impact of attentional focus strategies on the biomechanics of single-leg drop landing movements among individuals with FAI.

**Methods:**

Eighteen males with unilateral FAI were recruited. Kinematic and kinetic data were collected using an infrared three-dimensional motion capture system and force plates. Participants performed single-leg drop landing tasks under no focus (baseline), internal focus (IF), and external focus (EF) conditions. Biomechanical characteristics, including joint angles, ground reaction forces, and leg stiffness, were assessed. A 2 × 3 [side (unstable and stable) × focus (baseline, IF, and EF)] Repeated Measures Analysis of Variance (RM-ANOVA) analyzed the effects of attentional focus on biomechanical variables in individuals with FAI.

**Results:**

No significant interaction effects were observed in this study. At peak vertical ground reaction force (vGRF), the knee flexion angle was significantly influenced by attentional focus, with a markedly greater angle under EF compared to IF (*p* < 0.001). Additionally, at peak vGRF, the ankle joint plantarflexion angle was significantly smaller with EF than with IF (*p* < 0.001). Significant main effects of focus were found for peak vGRF and the time to reach peak vGRF, with higher peak vGRF values observed under baseline and IF conditions compared to EF (*p* < 0.001). Participants reached peak vGRF more quickly under IF (*p* < 0.001). Leg Stiffness (k_leg_) was significantly higher under IF compared to EF (*p* = 0.001).

**Conclusion:**

IF enhances joint stability in FAI, whereas EF promotes a conservative landing strategy with increased knee flexion, dispersing impact and minimizing joint stress. Integrating these strategies into FAI rehabilitation programs can optimize lower limb biomechanics and reduce the risk of reinjury.

## 1 Introduction

Ankle sprains are among the injuries most commonly sustained during sports activities (Medina McKeon and Hoch, 2019). They are especially prevalent during high-intensity actions such as leaping and drop landing, where the risk of injury is significantly increased to between 25 and 50 percent (Wang et al., 2025). If a sprained ankle is not properly managed and restored, it can lead to chronic discomfort and recurrent swelling, which are consequences that persist (Kong et al., 2023). Diminished movement control and an increased risk of recurrent injuries can result from this, potentially evolving into Functional Ankle Instability (FAI) (Cao et al., 2019). Joint stability and proprioception can be adversely affected by FAI, which in turn impairs motor performance and overall quality of life (Cain et al., 2020; Nunes et al., 2020).

In sports activities, drop landing tasks are particularly challenging and require substantial stability and coordination of the lower limbs (Meng et al., 2022). For individuals with FAI, performing drop landing tasks presents considerable challenges. This is due to impaired ankle joint stability and proprioception, which cause difficulties in controlling joint motion and force distribution, thereby consequently increasing the risk of injury (Xue et al., 2021). Previous research indicates that individuals with FAI demonstrate substantial differences between the stable and unstable limbs regarding joint angles and ground reaction forces during drop landing tasks, that are associated with motor control impairments and heightened injury risk (Zhang et al., 2014). Therefore, examining the biomechanical characteristics of individuals with FAI during drop landings, as well as the potential for enhancing lower limb stability and inherent coordination, is a primary focus in rehabilitation research.

Attention focus strategies constitute a significant concept in sports training and rehabilitation. Adjusting an individual’s attention distribution can markedly influence their motor execution efficiency and stability (Dalvandpour et al., 2021). Internal focus strategies (IF) typically direct attention towards an individual’s own body movements and sensations, whereas external focus strategies (EF) emphasize attention on the external environment and targets (Chua et al., 2021). Previous studies suggest that the EF strategy may diminish dependence on impaired proprioception and improve motor patterns and efficiency (An and Wulf, 2024). For individuals with FAI, employing an EF strategy significantly enhances motor control during drop landing. Moreover, the IF strategy effectively improves the internal perception of movement execution (Aiken and Becker, 2023), may support motor control and proprioception restoration in individuals with FAI, thereby improving motor performance and self-assurance. Although current research has demonstrated that attention focus strategies positively affect sports performance (Slovák et al., 2024), there remains a paucity of research regarding how modifying attention focus can optimize the biomechanics during drop landing movement of individuals with FAI.

Thus, this study aimed to examine the kinematic and kinetic attributes of the lower limbs during single-leg drop landings in individuals with unilateral FAI, under conditions of IF and EF. We advance the following hypotheses: 1) the IF and EF strategies will exert distinct influences on both the unstable and stable limbs of individuals with FAI; 2) the judicious use of focus strategies could potentially optimize the biomechanical characteristics during single-leg drop landings for individuals with FAI.

## 2 Methods

### 2.1 Participants

A total of 18 males with FAI participated in the study, and all provided informed consent. The Soochow University Ethics Committee Board approved this study (Table 1). The inclusion criteria for patients with FAI were as follows. 1) Participants must have a history of at least one unilateral ankle sprain within the past year and a sense of instability; 2) Participants should have no history of severe lower limb injury (Wu et al., 2021), except for ankle sprains; 3) CAIT score ≤24 (Hiller et al., 2007); 4) FAI symptoms are limited to the unilateral ankle joint. The exclusion criteria were as follows: 1) Participants with a history of sprains in both ankles (Wu et al., 2021); 2) Participants with acute pathological symptoms in the lower limbs; 3) Participants with a history of lower limb surgery (Wang et al., 2022); 4) Participants with congenital joint deformities; 5) Participants with a positive tilt of the talus or anterior drawer test results.

**TABLE 1:** Basic information of subjects ( $\bar{x}\;\pm \;S$ ).

Item	Experimental group (n = 18)
Age (years)	23.5 ± 1.7
Height (cm)	177.9 ± 6.3
Body mass index (kg/m^2^)	23.0 ± 2.0
Cumberland ankle instability tool score	18.8 ± 1.9
Unstable side (Left\Right)	(6\12)

### 2.2 Experimental procedure

**FIGURE 1:**
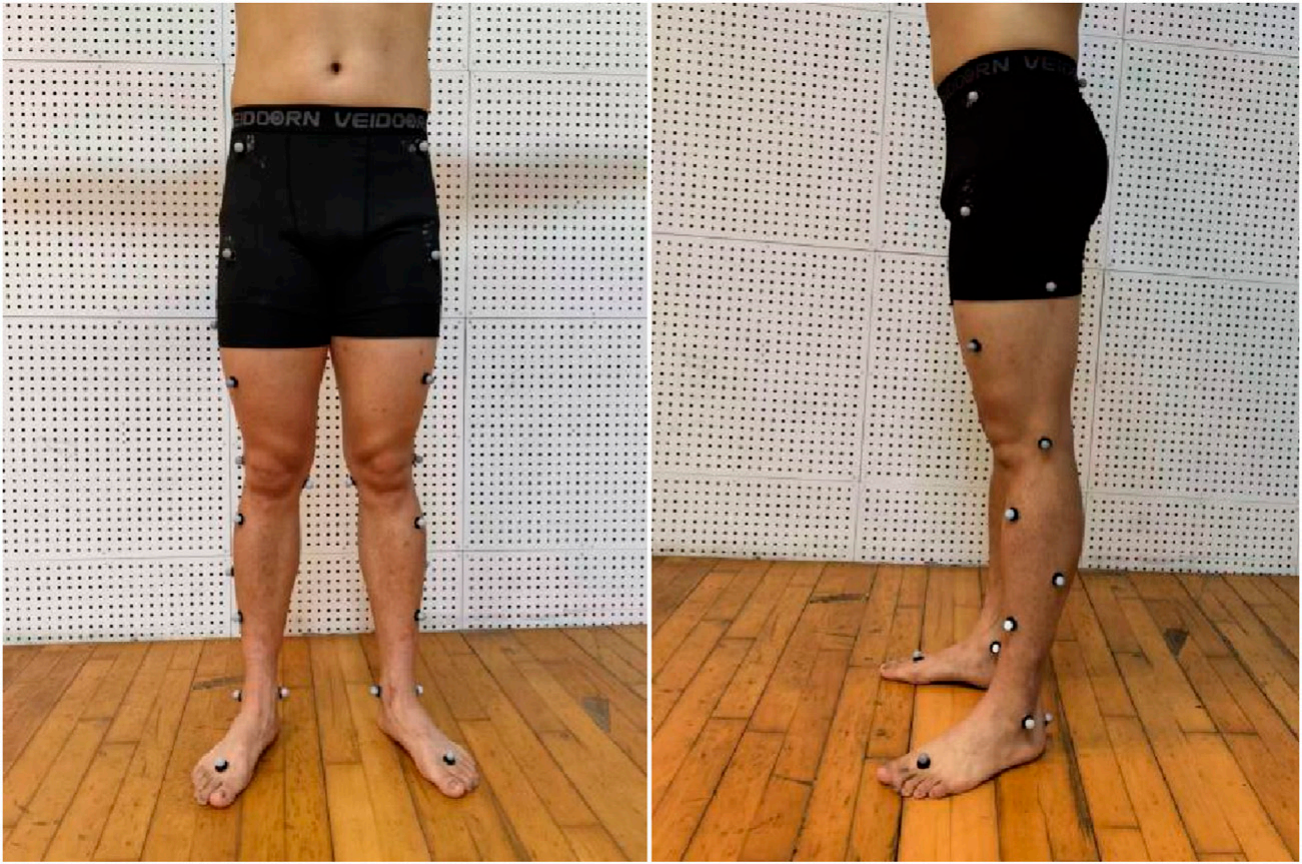
Marker points pasting diagram.

All participants wore standardized laboratory testing attire prior to the experiment to minimize the influence of external variables on the results. Participants initially engaged in adequate warm-up activities to prepare for subsequent testing. After the warm-up, researchers affixed 28 marker points with a diameter of 14 mm onto the participants to capture precise kinematic data (Figure 1).

Participants stood naturally on a 30 cm high jump box with feet shoulder-width apart, placing their hands on their waists during the test to prevent arm swing inertia. Following the baseline test, participants received verbal instructions for two different focus conditions.

In the IF condition, participants were instructed to focus on the action of “flexing the lower limb joints upon drop landing.” In the EF condition, they were instructed to “focus on achieving a reduced impact sound upon drop landing” (Almonroeder et al., 2020).

**FIGURE 2:**
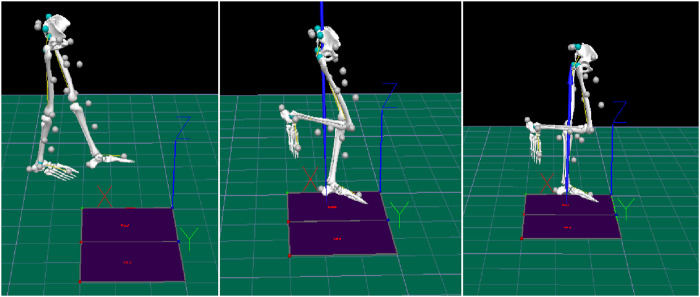
Action diagram. Participants performed a single leg drop landing from a 30-cm high platform onto a force plate.

All drop landing trials were conducted between conditions, with instructions given only once before the start of each condition to ensure consistency. Participants completed tests of single-leg drop landing tasks on both the stable and unstable sides under baseline, IF, and EF conditions. During the landing, participants were instructed to keep their non-supporting leg naturally bent to the side of their body. This posture was maintained to ensure a controlled and consistent landing motion (Figure 2). Under each test condition, participants were required to complete three successful single-leg drop landing movements, adhering to the specified landing posture. The results of the three tests for each participant were averaged to reduce random error and enhance the reliability of the data.

### 2.3 Data processing

Kinematic data were captured using a motion analysis system that comprised eight infrared cameras (Vicon Motion Analysis, United Kingdom) by tracking 28 infrared reflective balls (reflective markers) with a diameter of 14 mm at 100 Hz. The infrared reflective balls were stick to participants’ corresponding parts following the scheme suggested by the CGM 23 lower limb model. Kinetic data were capture using a 3D force plate (Kistler, Switzerland) at 1,000 Hz, which was synchronized with motion analysis system. Kinematic and kinetic data were firstly processed by Vicon Nexus 2.1.2. Both kinematic and kinetic data were then imported to Visual3D (Version 6, C-Motion, Inc., United States) for further processing. To enhance data quality, a fourth-order low-pass Butterworth filter was applied to smooth the three-dimensional coordinate data with a cutoff frequency set at 10 Hz; concurrently, the force platform data were filtered accordingly with a cutoff frequency of 50 Hz, and the calculation of lower limb joint angles was conducted using the Euler angle method (Yao et al., 2024). The IC was defined as the initial instance when vGRF exceeded 10 N (Christoforidou et al., 2017). The following data were analyzed (Meng et al., 2022): joint angles (°): joint angles of the hip, knee, and ankle in the sagittal and frontal planes at the initial contact (IC) and peak vertical ground reaction force (vGRF) moments; weight-normalized peak GRF [including vGRF, medial GRF (mGRF), and lateral GRF (lGRF)] (BW); Time to peak vGRF (s): This study recorded the time required for the vGRF to reach its peak from the IC; Leg Stiffness (kleg, BW/m), k_leg_ was calculated using Equation 1 (Zhang et al., 2017).

$$k_{\text{leg}}={\text{Fz}}_{\max}\;/\;\Delta L$$ (1)

Fz_max_ represents the peak vGRF, and ΔL represents the maximum length change of the lower limb during the drop landing process.

### 2.4 Statistical analysis

Data were analyzed using SPSS 26, with descriptive statistics presented as mean ± standard deviation ( $\bar{x}\;\pm \;S$ ). The normality of the data was tested using the Shapiro-Wilk test to ensure the applicability of subsequent analyses. A 2 × 3 [side (stable and unstable) × focus (baseline, IF, and EF)] repeated measures analysis of variance (RM-ANOVA) with Greenhouse-Geisser correction was conducted to assess the influence of different conditions on the outcomes. The criterion for statistical significance was set at α = 0.05. Post hoc analyses were conducted using the Bonferroni correction, with an adjusted adjusted *p*-value threshold of 0.017. Once an interaction effect be detected in the analysis, further simple effect analysis would be conducted to specify the source of the interaction effect. If no interaction effect is found, the main effects would be analyzed directly.

## 3 Results

At the IC, the main effect of attentional focus on the hip joint flexion angle was statistically significant (*F* = 8.777, *p* = 0.001, eta^2^ = 0.403). However, *post hoc* analyses with correction for multiple comparisons did not reveal significant differences between the baseline and either the IF or EF conditions (*p* > 0.017). At the peak vGRF, a similar pattern emerged, with a significant main effect of attentional focus observed (*F* = 7.401, *p* = 0.003, eta^2^ = 0.363), yet no statistically significant differences were detected after correction for multiple comparisons (*p* > 0.017) (Table 2).

**TABLE 2:** Hip joint angles at IC and peak vGRF moments during single drop landing.

Variables	Unstable side	Stable side	Main effect		Interaction effect
			Focus	Side	Focus × side
IC					
Hip joint flexion (+)/extension (−) angle (°)					
Baseline	16.83 ± 6.29	16.95 ± 3.95	*p* = 0.001*	*p* = 0.555	*p* = 0.804
IF	23.41 ± 9.47	24.54 ± 8.23			
EF	24.04 ± 9.83	24.44 ± 7.57			
Hip joint adduction (+)/abduction (−) angle (°)					
Baseline	−4.67 ± 4.60	−6.64 ± 2.65	*p* = 0.170	*p* = 0.206	*p* = 0.950
IF	−6.15 ± 4.04	−7.55 ± 3.77			
EF	−6.66 ± 4.80	−8.16 ± 3.99			
Peak vGRF					
Hip joint flexion (+)/extension (−) angle (°)					
Baseline	24.04 ± 8.60	23.14 ± 6.67	*p* = 0.003*	*p* = 0.787	*p* = 0.607
IF	29.49 ± 10.01	30.80 ± 9.01			
EF	33.10 ± 11.23	33.65 ± 8.65			
Hip joint adduction (+)/abduction (−) angle (°)					
Baseline	−3.01 ± 4.40	−5.33 ± 3.29	*p* = 0.108	*p* = 0.168	*p* = 0.885
IF	−5.28 ± 3.63	−6.86 ± 4.31			
EF	−5.14 ± 5.01	−6.49 ± 4.78			

*p* < 0.05*

At the peak vGRF, a significant main effect of attentional focus on knee flexion angle was observed (*F* = 7.127, *p* = 0.012, eta^2^ = 0.354). Specifically, *post hoc* analysis with Bonferroni correction indicated that compared to the IF condition, the EF condition exhibited a significantly greater knee flexion angle (*p* = 0.003). Concurrently, a significant main effect of side was observed in knee joint varus angle, with the unstable side showing a higher varus angle compared to the stable side (*F* = 6.119, *p* = 0.028, eta^2^ = 0.320) (Table 3).

**TABLE 3:** Knee joint angles at IC and peak vGRF moments during single drop landing.

Variables	Unstable side	Stable side	Main effect		Interaction effect
			Focus	Side	Focus × side
IC					
Knee flexion (+)/extension (−) angle (°)					
Baseline	10.18 ± 4.11	8.39 ± 3.47	*p* = 0.156	*p* = 0.881	*p* = 0.061
IF	11.68 ± 4.56	12.64 ± 4.85			
EF	11.58 ± 5.53	12.76 ± 5.19			
Knee varus (+)/valgus (−) angle (°)					
Baseline	0.85 ± 2.38	0.57 ± 3.04	*p* = 0.275	*p* = 0.322	*p* = 0.890
IF	1.85 ± 2.68	1.39 ± 3.07			
EF	2.30 ± 2.93	1.55 ± 3.06			
Peak vGRF					
Knee flexion (+)/extension (−) angle (°)					
Baseline	28.29 ± 6.61	27.16 ± 7.40	*p* = 0.012*	*p* = 0.746	*p* = 0.550
IF	28.67 ± 6.37	30.25 ± 6.64			
EF	36.94 ± 10.99	37.90 ± 11.86			
Knee varus (+)/valgus (−) angle (°)					
Baseline	1.44 ± 4.91	−1.46 ± 4.82	*p* = 0.778	*p* = 0.028*	*p* = 0.549
IF	2.89 ± 5.67	−1.92 ± 4.87			
EF	2.63 ± 7.38	−2.95 ± 6.61			

*p* < 0.05*

At the IC, a significant main effect of side was observed on the ankle joint inversion angle, with the unstable side demonstrating a higher inversion angle compared to the stable side (*F* = 15.337, *p* = 0.002, eta^2^ = 0.542); at the peak vGRF, a significant main effect of focus was noted on the ankle joint plantarflexion angle (*F* = 6.369, *p* = 0.017, eta^2^ = 0.329). Post hoc analysis with Bonferroni correction indicated that the EF condition showed a significantly smaller ankle joint plantarflexion angle compared to the IF condition (*p* < 0.001) (Table 4).

**TABLE 4:** Ankle joint angles at IC and peak vGRF moments during single drop landing.

Variables	Unstable side	Stable side	Main effect		Interaction effect
			Focus	Side	Focus × side
IC					
Ankle dorsiflexion (+)/plantarflexion (−) angle (°)					
Baseline	−18.35 ± 5.85	−18.58 ± 5.65	*p* = 0.378	*p* = 0.069	*p* = 0.082
IF	−18.64 ± 6.25	−15.60 ± 6.06			
EF	−21.47 ± 5.53	−17.75 ± 5.09			
Ankle inversion (+)/eversion (−) angle (°)					
Baseline	5.66 ± 3.82	2.80 ± 4.24	*p* = 0.312	*p* = 0.002*	*p* = 0.306
IF	7.42 ± 5.38	3.06 ± 3.53			
EF	8.28 ± 4.86	3.39 ± 3.31			
Peak vGRF					
Ankle dorsiflexion (+)/plantarflexion (−) angle (°)					
Baseline	11.15 ± 4.65	12.38 ± 6.11	*p* = 0.017*	*p* = 0.376	*p* = 0.982
IF	7.65 ± 5.35	8.38 ± 3.63			
EF	13.33 ± 4.52	14.22 ± 8.02			
Ankle inversion (+)/eversion (−) angle (°)					
Baseline	0.13 ± 3.39	−0.27 ± 5.25	*p* = 0.521	*p* = 0.823	*p* = 0.628
IF	0.84 ± 4.37	0.16 ± 3.45			
EF	−0.69 ± 4.72	−0.25 ± 3.45			

*p* < 0.05*

Significant main effects of focus were observed on the peak vGRF and the time to peak vGRF variables (*F* = 13.946, *p* < 0.001, eta^2^ = 0.518). Post hoc analysis with Bonferroni correction indicated that compared to the EF condition, participants under baseline and IF conditions exhibited a higher peak vGRF (*p* = 0.002, *p* < 0.001). Furthermore, under the IF condition, participants reached the peak vGRF in a significantly shorter time (*F* = 14.936, *p* < 0.001, eta^2^ = 0.535). For the index of k_leg_, the main effect of focus was also significant (*F* = 4.859, *p* = 0.016, eta^2^ = 0.272). Post hoc analysis with Bonferroni correction found that participants under the IF condition showed greater k_leg_ compared to the EF condition (*p* = 0.001). Additionally, the main effect analysis of side indicated a significant increase in k_leg_ among participants with the unstable side (*F* = 5.121, *p* = 0.041, eta^2^ = 0.283) (Table 5).

**TABLE 5:** Peak GRF and kleg during single drop landing.

Variables	Unstable side	Stable side	Main effect		Interaction effect
			Focus	Side	Focus × side
Peak vGRF (BW)					
Baseline	2.83 ± 0.64	2.42 ± 0.66	*p* < 0.001*	*p* = 0.078	*p* = 0.400
IF	2.99 ± 1.17	2.77 ± 0.86			
EF	1.73 ± 0.42	1.70 ± 0.47			
Peak mGRF (BW)					
Baseline	0.13 ± 0.02	0.12 ± 0.01	*p* = 0.445	*p* = 0.138	*p* = 0.306
IF	0.13 ± 0.04	0.13 ± 0.04			
EF	0.14 ± 0.02	0.11 ± 0.04			
Peak lGRF (BW)					
Baseline	−0.14 ± 0.02	−0.13 ± 0.02	*p* = 0.174	*p* = 0.821	*p* = 0.634
IF	−0.14 ± 0.04	−0.14 ± 0.05			
EF	−0.12 ± 0.03	−0.13 ± 0.03			
Time to Peak vGRF(s)					
Baseline	0.07 ± 0.01	0.07 ± 0.01	*p* < 0.001*	*p* = 1.000	*p* = 0.691
IF	0.06 ± 0.01	0.06 ± 0.02			
EF	0.09 ± 0.03	0.09 ± 0.03			
k_leg_ (BW/m)					
Baseline	19.79 ± 10.25	16.44 ± 9.83	*p* = 0.016*	*p* = 0.041*	*p* = 0.506
IF	16.58 ± 9.13	14.48 ± 7.40			
EF	10.08 ± 5.05	9.44 ± 4.23			

*p* < 0.05*

## 4 Discussion

### 4.1 The influence of attentional focus strategies on lower limb biomechanics in individuals with FAI

At the peak vGRF, individuals with FAI exhibited a significantly smaller knee flexion angle under IF conditions compared to EF conditions. The findings indicate that EF promotes a landing strategy that enhances shock absorption, beneficial for reducing impact on lower limbs. Higher knee flexion angles, associated with a “soft landing,” help disperse impact forces and protect joints (Laughlin et al., 2011; Wu et al., 2013). Given that individuals with FAI may have compromised stability in their lower limb joints (Liu et al., 2024), the increased knee flexion under EF conditions acts as an effective shock absorber, contributing to the reduction of peak impact forces through extended contact time and thus maintaining movement stability—a factor critical for joint protection and injury risk reduction. (Doherty et al., 2016). Furthermore, further analysis revealed that during the drop landing process, individuals with FAI exhibited a higher varus angle at the knee joint on the unstable side, which may be a compensatory biomechanical adjustment made to adapt to the compromised stability of the ankle joint (Jamaludin et al., 2020; Lima et al., 2020). This adjustment reflects a conservative strategy by individuals with FAI during landing. Furthermore, it facilitates effective force dispersion and absorption by increasing knee flexion (Blackburn and Padua, 2008). It is noteworthy that the increase in the varus angle is not mutually exclusive with the conservative drop landing strategy; rather, they complement each other, jointly promoting the optimization of the lower limb’s biomechanical response. Moreover, this adjustment of the varus angle may help individuals with FAI maintain balance under unstable conditions, serving as an effective means of dispersing influence forces. Tamura et al. (2017) suggest that an appropriate varus angle at the knee during unilateral drop landing tasks can better absorb and cushion the shock, underscoring the importance of this strategy for managing joint stress. From the perspective of biomechanical adjustment theory that the rigid control associated with IF may increase joint stress, while the flexible control under EF conditions can improve impact dispersion, helping to reduce peak ground reaction forces and thereby protect the joints (Wang et al., 2023). Therefore, this finding from the study emphasizes the need to pay special attention to the stability and control ability of the unstable lower limb in the rehabilitation training of individuals with FAI.

Additionally, this study found that individuals with FAI exhibited a greater varus angle at the ankle joint on the unstable side during drop landing, potentially linking to the impaired stability of the ankle joint (Simpson et al., 2019). At the peak vGRF, the plantarflexion angle of the ankle joint under EF conditions was smaller, indicating that the EF strategy might contribute to a more stable ankle joint position during landing, which could indirectly lower the risk of injury. Furthermore, from a neuromuscular control perspective, the EF strategy may promote a more focused landing approach by directing attention to the external environment, potentially enhancing stability. rather than their body movements (Mulla and Keir, 2023), reducing reliance on the compromised stability of the ankle joint and promoting more natural whole-body coordination, which is crucial for optimizing motor control and reducing injury risk (Singh et al., 2022). The EF strategy, by directing the attention of individuals with FAI to the external environment rather than their body movements, may encourage them to adopt more stable and effective movement patterns. This is consistent with the findings of Bæktoft van Weert et al. (2023), which suggest that the EF strategy can lead to improved jumping and drop landing techniques. Similarly, Widenhoefer et al. (2019) argue that training with an emphasis on an EF fosters adaptation of the body’s drop landing mechanisms. This strategy not only helps to optimize the movement control of the ankle joint but may also enhance the overall stability and coordination of the lower limbs. Therefore, incorporating the EF strategy in the design of rehabilitation training programs is of significant importance for enhancing the softness and stability of landing in individuals with FAI. By adjusting the focus of attention, the EF strategy may assist patients in achieving softer and more stable landings, reducing excessive motion and lowering the risk of injury.

The overall findings suggest that attentional focus strategies exert a significant influence on the biomechanics of the lower limbs among individuals with FAI, notably by promoting softer and more stable landings. The study, however, did not identify any interactive effects, implying that the impact of these strategies on both unstable and stable lower limb joints is consistent, which may indicate that individuals with FAI may benefit from the adjustment of attentional focus strategies during drop landing movements, regardless of whether it is the unstable or stable side.

### 4.2 Optimization of drop landing stability in individuals with FAI through attentional focus strategies

This study observed significant main effects of focus on both peak vGRF and the time to peak vGRF. Although individuals with FAI under baseline and IF conditions exhibited higher peak vGRF compared to the EF strategy, the time to reach peak vGRF was significantly reduced under the IF condition. Moreover, under the IF condition, individuals with FAI demonstrated greater k_leg_, with a significant increase on the unstable side in terms of k_leg_, indicating that the IF strategy may aid in enhancing the stability and support capacity of the lower limbs, reducing unstable elements during the drop landing process, thereby diminishing the injury risk for individuals with FAI during physical activity. However, under the EF strategy, individuals with FAI exhibited a longer duration to reach peak vGRF, attributable to the significant increase in knee flexion angle under the EF strategy. As the knee flexion angle increases, the influence force during drop landing is more effectively dispersed and absorbed, thereby extending the contact time with the ground (Harry et al., 2019). This extended contact time allows patients to adjust the posture of their lower limbs by increasing knee flexion, enabling a more effective cushioning against the influence from the ground, thereby augmenting the time to reach the peak vGRF (Almonroeder et al., 2020). The EF strategy, by prompting individuals with FAI to focus on the external environment rather than bodily sensations (Chua et al., 2021), may assist individuals with FAI in controlling the drop landing motion more gracefully, achieving a smoother force transmission process. Under this strategy, increasing the knee flexion angle not only aids in shock absorption but also provides patients with more time to adjust the posture of their lower limbs, refine the drop landing motion, thereby reducing the instantaneous influence on the lower limb joints. Therefore, the extended peak vGRF time under the EF strategy reflects a more cautious and controlled drop landing approach, which is a safer and more effective sports strategy for individuals with FAI, consistent with the aforementioned discussion.

Attentional focus strategies may improve landing techniques in individuals with FAI by optimizing biomechanical responses related to softness and stability (Aiken and Becker, 2023). In particular, the EF strategy can better optimize drop landing stability (Vaz et al., 2019), and by reducing excessive activity of the ankle joint, may help to enhance the stability of the ankle joint and reduce the risk of sprains. Furthermore, the IF strategy, by prompting individuals with FAI to focus on the execution of drop landing movements, may help to reduce the influence force experienced by the joints and enhance the stability of movement. However, it should be noted that although the peak vGRF is higher under the IF condition, this does not necessarily imply an increased risk of injury; movements under this condition may require more attention to be allocated to internal bodily sensations, which could focus attention on movement execution and thereby enhance control and stability (Chen et al., 2023). Moreover, the time for individuals with FAI to reach peak vGRF is shortened under the IF condition, which may help to reduce unstable factors during the drop landing process, thereby enhancing stability. Additionally, increased kleg may help to provide better support and stability, further reducing the risk of injury. This finding is inconsistent with Wulf’s traditional constraint-led action hypothesis (Wulf et al., 2001a; Wulf et al., 2001b), which suggests that IF would constrain the motor system by interfering with the automation of movement regulation, while EF might allow the motor system to self-organize more naturally, without interference from conscious control, leading to more effective performance and learning. However, according to the results of this study, both IF and EF strategies have produced positive effects on the movement performance of individuals with FAI. Therefore, this hypothesis may not be applicable to individuals with FAI, possibly because the ankle joint injury in this population leads to a lack of lower limb stability and support (Yin et al., 2016), making them more focused on the execution of movements when adopting the IF strategy, thereby improving movement precision and control.

Based on the aforementioned perspectives, the results of this study suggest that the EF strategy may encourage individuals with FAI to adopt a more conservative drop landing strategy, achieving a “soft landing” by increasing the knee flexion angle, thereby reducing direct influence and pressure on the knee joint. Furthermore, the EF strategy may also help to reduce excessive activity of the ankle joint and enhance its stability. At the same time, the IF strategy may help individuals with FAI to focus more on the execution of their movements upon drop landing, thereby improving the stability and support capacity of the lower limbs. These findings support Hypothesis 2 of this study, that appropriate attentional focus strategies have a positive effect on improving the biomechanical characteristics and motor control of individuals with FAI. However, both IF and EF strategies have certain drawbacks; while the IF strategy may enhance stability, in some cases, excessive IF might increase muscle tension, thereby affecting the fluidity and naturalness of movement. The EF strategy could direct attention away from the execution details, potentially diminishing the fluidity of movements and impacting overall stability. Therefore, in rehabilitation training, the combined use of IF and EF strategies can enhance the adaptability and flexibility of patients. For instance, employing the IF strategy to establish correct movement patterns in individuals with FAI; utilizing the EF strategy to enhance their adaptability to the external environment. Furthermore, a tailored approach to selecting or integrating IF and EF strategies, customized to the unique conditions and rehabilitative aspirations of each individual with FAI, may represent the most efficacious strategy.

### 4.3 Clinical recommendations

This study elucidates the importance of employing diverse attentional focus strategies within personalized and comprehensive rehabilitation programs for individuals with FAI. When devising personalized rehabilitation plans for individuals with FAI, it is recommended that the IF strategy be employed for those who require the establishment of proper movement patterns and enhancement of lower limb stability; For those with FAI who need to optimize their adaptability to the external environment and the fluidity of movement, the EF strategy is recommended. By integrating IF and EF strategies, an efficacious rehabilitation training regimen can be crafted for individuals with FAI, enhancing movement execution, reducing injury risk.

### 4.4 Limitations

In this study, significant main effects of attention focus on hip joint flexion angle were observed at the IC and at the peak vGRF. Specifically, compared with the baseline, both IF and EF conditions demonstrated a trend of change in hip joint flexion angle. However, after Bonferroni correction, these changes did not reach statistical significance. This may imply that although different attention focus strategies have an influence on the hip joint flexion angle, the influence is not statistically robust and may be interfered with by sample characteristics, test conditions, or other uncontrolled variables.

## 5 Conclusion

The tailored application of IF and EF strategies exerted distinct influences on biomechanical outcomes in individuals with FAI. The IF, by directing attention to body movements, enhanced lower limb stability and support capabilities, which is crucial for reducing landing influence and improving joint shock absorption. Conversely, the EF, which diverts attention away from body sensations, encouraged a more conservative landing strategy characterized by increased knee flexion angles. This approach not only facilitated a softer landing by effectively dispersing impact forces over a longer contact time but also helped in minimizing the instantaneous stress on the lower limb joints. Collectively, integrating these strategies into FAI rehabilitation programs can optimize lower limb biomechanics and reduce the risk of reinjury.

## Data Availability

The original contributions presented in the study are included in the article/supplementary material, further inquiries can be directed to the corresponding author.
